# Weighted gene co-expression network analysis revealed the key pathways and hub genes of potassium regulating cotton root adaptation to salt stress

**DOI:** 10.3389/fpls.2023.1132877

**Published:** 2023-03-01

**Authors:** Feiyan Ju, Liyuan Sun, Cai Xiong, Zhuo Wang, Huilian Yu, Jiali Pang, Hua Bai, Wengqing Zhao, Zhiguo Zhou, Binglin Chen

**Affiliations:** ^1^ College of Agriculture, Nanjing Agricultural University, Nanjing, China; ^2^ Collaborative Innovation Center for Modern Crop Production co-sponsored by Province and Ministry, Nanjing, China; ^3^ School of Agricultural Sciences, Northwest Missouri State University, Maryville, MO, United States

**Keywords:** WGCNA, salt stress, potassium, cotton, hub genes

## Abstract

Soil salinization is one of the main abiotic stresses affecting cotton yield and planting area. Potassium application has been proven to be an important strategy to reduce salt damage in agricultural production. However, the mechanism of potassium regulating the salt adaptability of cotton has not been fully elucidated. In the present research, the appropriate potassium application rate for alleviating salt damage of cotton based on different K^+^/Na^+^ ratios we screened, and a gene co-expression network based on weighted gene co-expression network analysis (WGCNA) using the transcriptome data sets treated with CK (0 mM NaCl), S (150 mM NaCl), and SK8 (150 mM NaCl + 9.38 mM K_2_SO_4_) was constructed. In this study, four key modules that are highly related to potassium regulation of cotton salt tolerance were identified, and the mitogen-activated protein kinase (MAPK) signaling pathway, tricarboxylic acid (TCA) cycle and glutathione metabolism pathway were identified as the key biological processes and metabolic pathways for potassium to improve cotton root salt adaptability. In addition, 21 hub genes and 120 key candidate genes were identified in this study, suggesting that they may play an important role in the enhancement of salt adaptability of cotton by potassium. The key modules, key biological pathways and hub genes discovered in this study will provide a new understanding of the molecular mechanism of potassium enhancing salinity adaptability in cotton, and lay a theoretical foundation for the improvement and innovation of high-quality cotton germplasm.

## Introduction

Soil salinization seriously threatens the sustainable development of global agriculture and ecological balance, leading to the shortage of food and bioenergy, and has become one of the serious environmental challenges that cannot be ignored (Zelm et al., 2020; Zhao et al., 2020). Currently, more than 1.125 billion hectares of land worldwide have been affected by salinity, and about 4.03 billion people have been threatened by crop cultivation due to soil salinity (Omuto et al., 2020). Therefore, promoting the development and utilization of saline-alkali land is of great practical significance for implementing sustainable development strategy and ensuring stable agricultural production.

Cotton (*Gossypium hirsutum* L.) is not only an important fiber crop and oil crop but also a raw material for the textile and fine chemical industry, which occupies a pivotal position in world economic development (Dong et al., 2020). The salt tolerance threshold of cotton is 7.7 dS m^-1^, which is considered as a pioneer crop for saline alkali land improvement (Zhang et al., 2013; Sharif et al., 2019). However, the increasing salt stress has seriously threatened the growth, yield and fiber quality of cotton (Peng et al., 2016; Ju et al., 2021), and hindered the sustainable production of cotton worldwide. Given that Na^+^ and K^+^ have similar ionic structural characteristics, a large number of Na^+^ will compete for the binding sites of K^+^ under salt stress, leading to K^+^ deficiency (Zhu, 2003; Shabala and Cuin, 2008). Therefore, appropriate increased application of potassium fertilizer is one of the important strategies to reduce salt damage (Pandey and Mahiwal, 2020; Mostofa et al., 2022). Contemporary studies have shown that the application of appropriate doses of potassium (K) can improve the resistance of plants to high salinity (Assaha et al., 2017; Kumar et al., 2020). K^+^ has been proved to alleviates the negative effects of salt stress by balancing ion homeostasis, controlling osmotic balance and scavenging reactive oxygen species (ROS) (Shabala and Cuin, 2008; Ahmad et al., 2016; Chakraborty et al., 2016; Parveen et al., 2020). Understanding the response and defense mechanism of cotton to salinity and exploring the key genes of potassium regulating cotton salt adaptability can provide theoretical support for optimizing cotton salt-tolerant variety breeding and improving cotton production efficiency.

Stress adaptation in plants is the result of the synergistic effects of multiple genes and metabolic pathways (Kobayashi et al., 2016). Therefore, identifying plant salt tolerance genes and mining good crop resources at the whole genome level is a key step to improve plant salt tolerance (Du et al., 2016). Several salt tolerance genes have been verified to regulate gene transcription, signal transduction and metabolic processes in plants (Zhu et al., 2019). However, so far, the achievements in the cultivation of plant salt-tolerant varieties are still very limited. There are still many genes with high potential that have not been discovered in the regulation of salt tolerance in cotton. The popularity of sequencing technology has generated massive omics data, which have been applied to the research of cotton stress resistance (Peng et al., 2014; Fan et al., 2021). As a kind of molecular biological network (Langfelder and Horvath, 2008), weighted gene co-expression network analysis (WGCNA) has been widely used in constructing gene interaction relationships, identifying key regulatory genes and predicting the function of unknown genes (Zhang and Horvath, 2005; Ruan et al., 2010; Wan et al., 2018). Based on WGCNA, previous studies on the salt tolerance response of cotton (Xu et al., 2020), maize (*Zea mays* L.) (Li et al., 2021; Ma et al., 2021), rice (*Oryza sativa* L.) (Zhu et al., 2019), wheat (*Triticum aestivum* L.) (Wang et al., 2022), Arabidopsis (*Arabidopsis thaliana*) (Kobayashi et al., 2016), bermudagrass (*Cynodon dactylon* (L.) Pers.) (Shao et al., 2021) and green halophytic microalgae (*Dunaliella salina*) (Panahi and Hejazi, 2021) were conducted, and the key regulatory pathways and hub genes of salt adaptation in plants were identified, which provided useful information for further exploration of the transcriptional regulation mechanism of salt tolerance in plants.

The root is the initial and most sensitive organ to sense salt damage in plants (Munns and Tester, 2008; Coudert et al., 2010), and is also an ideal target to study the molecular mechanism of salt tolerance in plants (Shao et al., 2021). To date, some salt-responsive transcriptome studies have been conducted in cotton roots (Wei et al., 2017; Guo et al., 2019; Wang et al., 2020; Xu et al., 2020). However, there are relatively few reports on the transcriptional regulation of potassium on cotton root adaptation to salt stress. How potassium improves salt tolerance of cotton through transcriptional regulation and what key regulatory factors in this process have not been clearly explained. However, salt adaptation of plants is a complex quantitative trait controlled by multiple genes, and the screening of hub genes regulating salt tolerance has always been an important challenge in cotton genetics and breeding.

Here, we carried out WGCNA based on a gene data set obtained from RNA-Seq to explore the key functional modules and biological metabolic pathways of potassium regulating cotton root adaptation to salt stress, identify the salt stress response hub genes and candidate genes, understand the molecular mechanisms of potassium improving cotton salt adaptability, and provide insights for the breeding of salt-tolerant cotton varieties.

## Materials and methods

### Plant materials and experimental treatment

The salt-tolerant variety “Zhong9807” was bred by the Cotton Research Institute, Chinese Academy of Agricultural Sciences, with a salt-tolerance index of 51.22 and a growth period of approximately 116 days. The experiment was carried out in the intelligent greenhouse of Nanjing Agricultural University. Seedlings were incubated in quartz sand and transferred to 1/8 Hoagland nutrient solution when the first true leaf expanded. The following treatments were carried out in the 2-leaf stage of cotton: CK (0 mM NaCl), S (150 mM NaCl), SK16 (150 mM NaCl + 4.96 mM K_2_SO_4_), SK8 (150 mM NaCl + 9.38 mM K_2_SO_4_), SK4 (150 mM NaCl + 18.76 mM K_2_SO_4_), SK2 (150 mM NaCl + 37.52 mM K_2_SO_4_), SK1 (150 mM NaCl + 75.04 mM K_2_SO_4_), (i.e., K^+^/Na^+^ = 1/16, 1/8, 1/4, 1/2, 1/1, respectively). Three biological replicates were set for each treatment, with 15 plants in each replicate, for a total of 315 plants. The culture medium was replaced every 5 days, oxygen was supplied throughout the day, the indoor temperature was (28 ± 1) °C/(24 ± 1) °C, the humidity was 50% ~ 65%, and the light source was natural light + LED light for 13 h/d.

### Sample collection and acquisition of transcriptome data

Cotton roots were collected at 1 h, 6 h, 12 h and 24 h after treatment for RNA sequencing. Each treatment contained 3 biological repeats, and 3 cotton plants were selected for each repeat. Transcriptome sequencing was carried out by Guangzhou Genedenovo Biotechnology Co., Ltd., and 79218 gene expression data were obtained from 36 samples.

### Physiological parameter measurements

The relative water content (RWC) of cotton leaves was calculated according to the drying weighing method. Leaf water potential was measured by a WP4C Dewpoint Potentia Meter. The relative conductivity (REC) was measured by a conductivity meter (DDS-307A, China). The contents of malondialdehyde (MDA) was determined according to Wang et al. (2022). According to the procedure described by Yang et al. (2017), the contents of K^+^ and Na^+^ in roots were determined by atomic absorption spectrophotometer (PinAAcle 900 T, America).

### Construction of the weight gene co-expression network

The co-expression network was constructed in R using the WGCNA (v1.47) package. The genes expressed as 0 in each sample were deleted, and then high-quality genes were screened according to the standard of the expression level ≥ 8 in at least one sample. According to the principle of a scale-free network, the appropriate power value was selected as the analysis parameter, and the average gene connectivity under different power values was calculated.

The gene clustering tree was constructed according to the correlation of gene expression, and then the genes with similar expression patterns were classified into the same module, and the branches of the cluster tree were cut and distinguished to produce different modules. The modules were merged with the threshold value of module eigenvalue similarity > 0.75, and the minimum gene number of modules was set as 50.

### Functional annotation and enrichment analysis of module genes

The GO and Pathway enrichment analysis provides all GO terms and metabolic/signal transduction pathways that are significantly enriched in module genes compared with the background genome and screened out module genes corresponding to biological functions. The genes in each module were mapped to the Gene Ontology database (http://www.geneontology.org/) and Kyoto Encyclopedia of Gene and Genome (KEGG) for functional and pathway enrichment analysis.

### Identification and analysis of vital modules and hub genes

The module eigenvalue represents the weighted comprehensive value of the expression of all genes in the module. Modules that are significantly correlated with specific samples are identified according to the heatmap of the sample expression patterns. In this study, modules with opposite gene expression patterns under SK8 treatment and S treatment were selected as vital modules. The vital pathways with significant enrichment (Q value ≤ 0.05) and related to the plant salt tolerance response were screened according to the KEGG Pathway enrichment results of the module, and the top 5 genes with gene connectivity were identified as hub genes.

### Construction of a regulatory network for hub genes and their associated candidate genes

The top 10 genes with connectivity to hub genes were considered as associated genes. Cytoscape_3.3.0 software was used to draw the co-expression network. Nodes in the network represent genes, and edges represent regulatory relationships between genes. The more connected genes there are, the more important the gene is in the interaction network.

### Validation of the DEGs by RT-qPCR

Twelve highly expressed hub genes were screened for qRT-PCR validation. Total RNA was extracted from the same samples that were used for sequencing. First-strand cDNA was synthesized using a HiScript III RT SuperMix for qPCR (+gDNA wiper) (Vazyme Biotech Co., Ltd, Nanjing, China). The primer sequences used were designed with a free online primer design tool (https://www.ncbi.nlm.nih.gov/) and synthesized by Tsingke Biotechnology (Beijing) Co., Ltd. The detail of primers was shown in the **Table S1**. RT-qPCR was performed on a CFX Connect (TM) Real-Time PCR Detection System (BIO-RAD, Singapore) using Taq Pro Universal SYBR Qpcr Master Mix (Vazyme Biotech Co., Ltd, Nanjing, China) according to the instructions. The cotton Actin gene was used as the endogenous control. Gene expression levels were calculated from the threshold cycle according to 2^-ΔΔCt^ (Livak and Schmittgen, 2001) and standard deviation was calculated between three biological replicates.

### Statistical analysis

Analysis of variance (ANOVA) was performed using the IBM SPSS statistical package (version 22.0) (IBM Inc., Ammonk, N.Y., USA). Treatment means were separated using the least significant difference (LSD) method at the P = 0.05 level. The bar graphs were drawn by the OriginPro 2021 software program (OriginLab Inc., Northampton, Massachusetts., USA).

## Results

### Phenotypic difference of cotton under different potassium application rates

**Figure 1:**
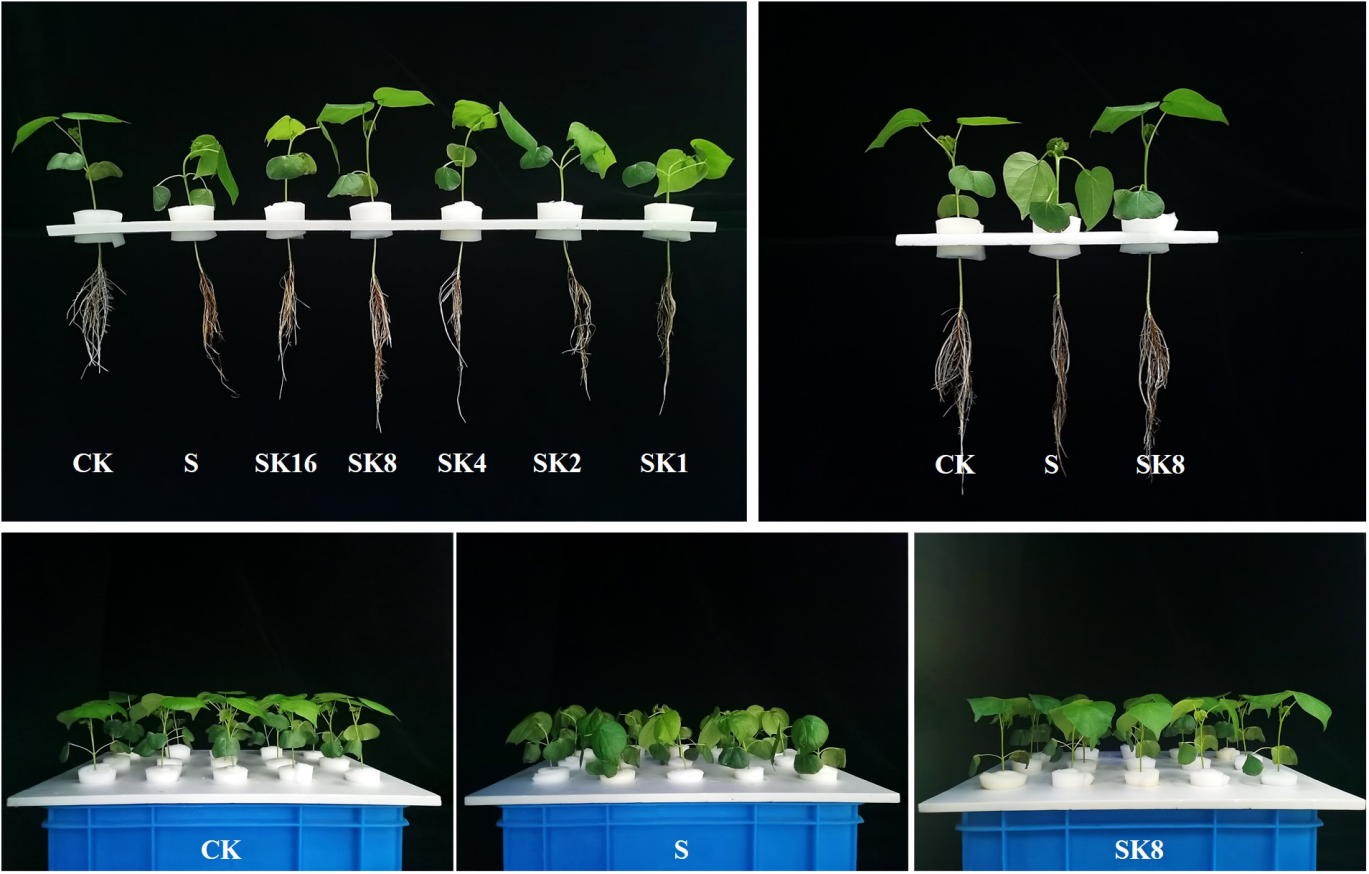
Phenotypic difference of cotton under different potassium application rates.

According to the phenotype of cotton leaf wilting symptoms under different potassium application rates (**Figure 1**), the study found that SK16 treatment did not alleviate the symptoms of cotton seedling wilting symptoms, SK4, SK2, SK1 treatment aggravated the symptoms of salt stress induced wilting symptoms, and with the increase of potassium application amount of cotton leaf wilting degree gradually worsened. After SK8 treatment for 1 h, cotton wilting symptoms began to improve, and the improvement effect was prominent at 6 h. By 24 h treatment, cotton leaf wilting symptoms had basically improved.

### Effects of different potassium application rates on physiological indexes of cotton

**Figure 2:**
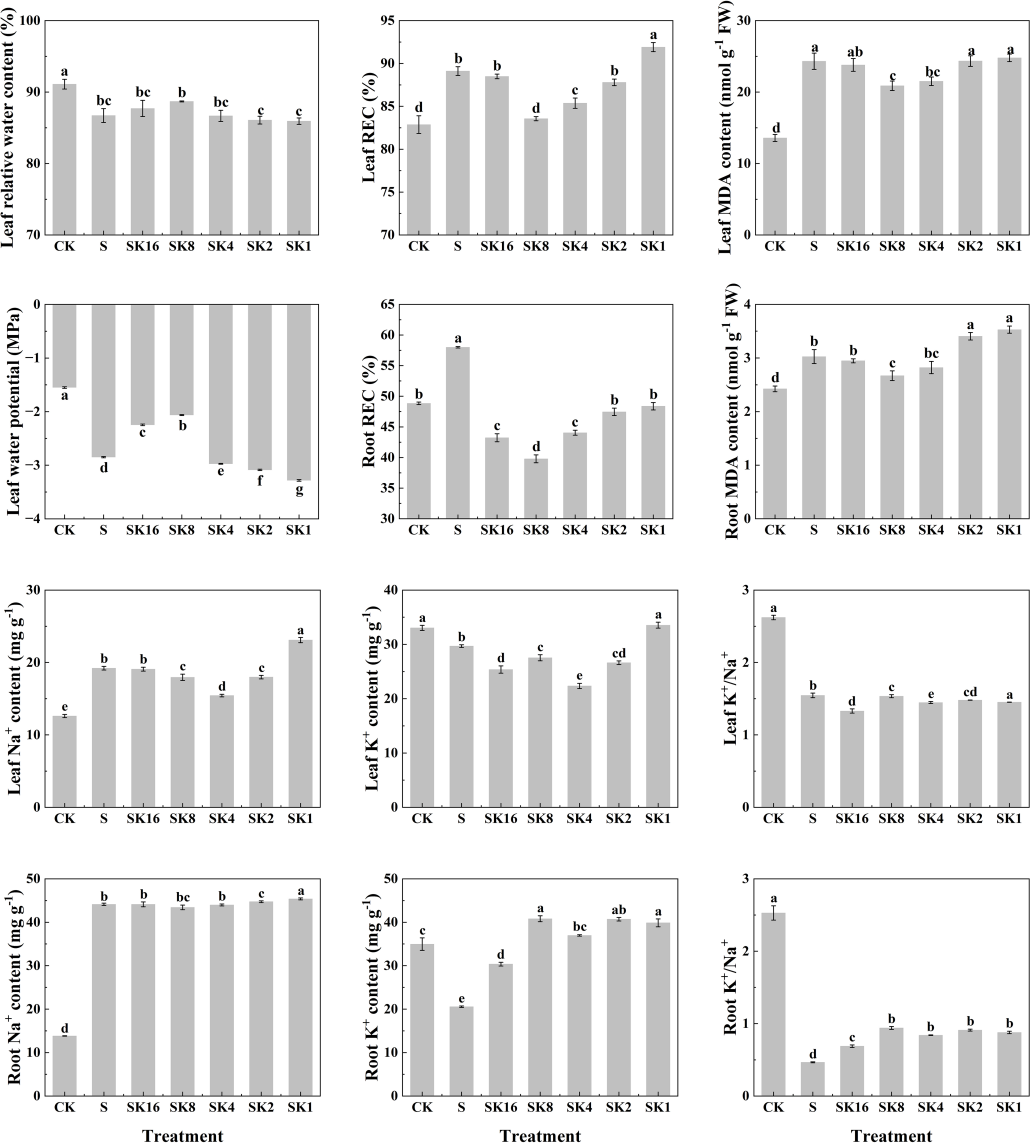
Effects of different potassium application rates on physiological indexes of cotton. REC, relative conductivity; MDA, malondialdehyde.

As shown in **Figure 2**, compared with S treatment, the relative water content and water potential of cotton leaves both increased first and then decreased with the increase of potassium application rates. The relative electrical conductivity of cotton leaves under SK16, SK8, SK4 and SK2 treatments decreased by 0.70%, 6.22%, 4.20% and 1.48%, respectively, while that under SK1 treatment increased by 3.14%. Furthermore, potassium application significantly decreased the relative electrical conductivity of cotton roots under salt stress, and the relative electrical conductivity of cotton roots under SK16, SK8, SK4, SK2 and SK1 treatments decreased by 25.47%, 31.40%, 24.06%, 18.18% and 16.59%, respectively, compared with S treatment. In addition, malondialdehyde content in cotton leaves and roots decreased first and then increased with the increase of potassium application. Specifically, compared with S treatment, leaf malondialdehyde content under SK16, SK8 and SK4 treatments decreased by 2.14%, 14.13% and 11.53%, respectively, and increased by 0.12% and 1.95% under SK2 and SK1 treatments, respectively. The root malondialdehyde content decreased by 2.48%, 11.68% and 6.65% under SK16, SK8 and SK4 treatments, respectively, while increased by 12.67% and 16.74% under SK2 and SK1 treatments.

Compared with S treatment, the leaf Na^+^ content decreased by 6.56%, 19.64% and 6.41% under SK8, SK4 and SK2 treatments, respectively, while increased by 20.23% under SK1 treatment. The root Na^+^ content under SK2 and SK1 treatments was increased by 1.35% and 2.82%. The K^+^ content of cotton leaves under SK16, SK8, SK4 and SK2 treatments was reduced by 14.52%, 7.23%, 24.71% and 10.31%, respectively, while that under SK1 treatment was significantly increased by 12.98%. Furthermore, the K^+^ content of cotton root under SK16, SK8, SK4, SK2 and SK1 treatment was increased by 47.25%, 99.19%, 79.87%, 89.33% and 94.61%, respectively. In addition, compared with S treatment, K^+^/Na^+^ of cotton leaves under SK16, SK4 and SK1 treatments significantly decreased by 13.97%, 6.40% and 6.08%, respectively. The root K^+^/Na^+^ of cotton under SK16, SK8, SK4, SK2 and SK1 treatment was significantly increased by 47.85%, 101.72%, 80.26%, 95.28% and 88.41%, respectively. In conclusion, combined with the changes of phenotypic and physiological indexes of cotton seedlings under various treatments, this study believes that K^+^/Na^+^=1/8 (i.e. SK8) ratio of potassium application had the best alleviating effect on salt damage of cotton. Therefore, transcriptome sequencing and WGCNA analysis were conducted based on CK, S and SK8 treatments in subsequent studies.

### Construction of the co-expression network by WGCNA

**Figure 3:**
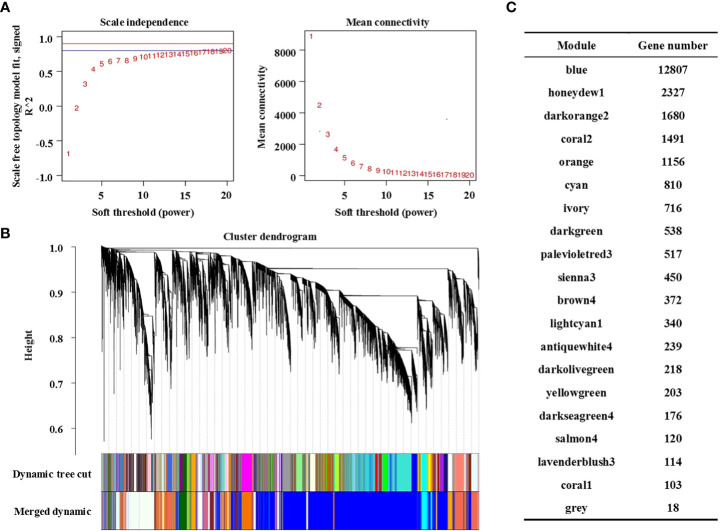
Construction of the co-expression network by WGCNA. **(A)** Soft power curve, the abscissa represents the power value, the ordinate (left) represents the correlation coefficient, and the ordinate (right) represents the average connectivity of genes; **(B)** Gene cluster dendrograms and module division; **(C)** Distribution of the number of genes in co-expression modules.

WGCNA is an unsupervised analysis method based on gene expression clustering. In this study, the data sets of 79218 genes obtained from RNA sequencing of the 36 samples mentioned above were analysed. To improve the stability of network construction, genes expressed as 0 in each sample were deleted first, and then high-quality genes were screened according to the standard of the expression level ≥ 8 in at least one sample. Finally, 24395 high-quality genes were obtained for WGCNA (**Table S2**). The WGCNA packet was used to calculate the weight values to make the network conform to the principle of scale-free network distribution. In this study, the minimum power value (β = 8) when the correlation coefficient reached the platform period was selected as the analysis parameter (**Figure 3A**, left), and the change in average gene connectivity under different power values was calculated (**Figure 3A**, right).

The gene clustering tree was constructed according to the correlation of gene expression. As shown in **Figure 3B**, a branch of the tree corresponds to a cluster of gene sets with highly correlated expression levels. The gene modules were divided according to the clustering relationship between genes. Genes with similar expression patterns were classified into the same module, and the branches of the clustering tree were cut and distinguished to produce different modules. Each color represents a module, and gray represents genes that cannot be classified into any module. After preliminary module division, the result of preliminary module division was obtained. Then, according to the threshold value of module eigenvalue similarity > 0.75, the modules with similar expression patterns were merged to obtain the final 20 co-expression modules. The number of genes contained in each module is shown in **Figure 3C**, in which the Blue module contains the largest number of genes (12807 genes) and the Coral1 module contains the least number of genes (103 genes). Details of genes in each module are shown in **Table S3**.

### Analysis of sample expression patterns and screening of key modules

**Figure 4:**
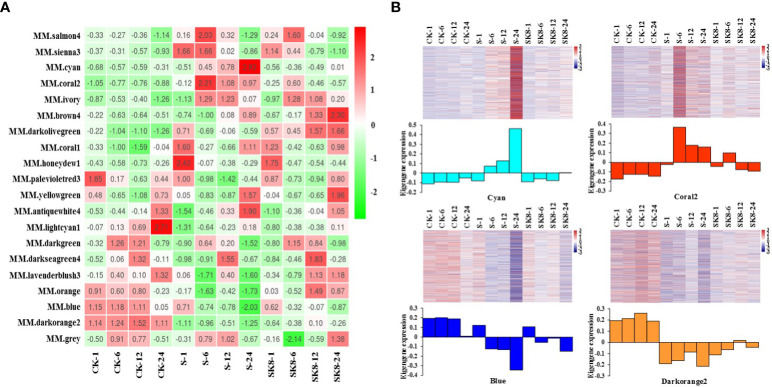
Sample expression heatmap of co-expression modules. **(A)**, the color in the heatmap indicates the characteristic value of the module, red represents high expression, and green represents low expression; **(B)**, the above figure is the expression heatmap of genes in the module in different samples, and the figure below shows the eigenvalues of modules in different samples, red indicates upregulation and blue indicates downregulation.

The expression mode of the module gene in each sample was displayed with module eigenvalue. The heatmap of the sample expression mode contributes to screening out the modules that are significantly related to specific samples to select corresponding modules for further research. The module eigenvalue is the weighted comprehensive value of the expression of all genes in the module, reflecting the comprehensive expression level of all genes in each sample. In this study, modules with significantly different expression patterns among different treatments were screened. As shown in **Figure 4A**, the Cyan module and Coral2 module showed the expression pattern of increased first and then decreased between CK-S-SK8 treatments. In contrast, the Blue module and Darkorange2 module showed the expression pattern of first decreasing and then increasing between CK-S-SK8 treatments. However, the expression mode of genes in the other modules was not obvious among the treatments. Therefore, this study identified the 4 co-expression modules as key modules for further analysis.

Analysis of the expression mode of genes showed that there was significant specificity in the expression of genes in each key module; specifically, genes in each module were significantly differentially expressed at the relevant treatment time points of the module (**Figure 4B**). For example, the genes in the Cyan module were significantly up-regulated at 24 h after salt treatment, the genes in the Coral2 module were significantly up-regulated at 6 h after salt treatment, and the genes in the Blue and Darkorange2 modules were significantly down-regulated at 24 h after salt treatment. In general, the expression of genes in the Cyan module and Coral2 module increased under salt stress but was inhibited under potassium application. However, the expression of genes in the Blue and Darkorange2 modules was inhibited under salt stress and significantly increased after potassium application.

### GO and KEGG enrichment analysis of genes in key modules

**Figure 5:**
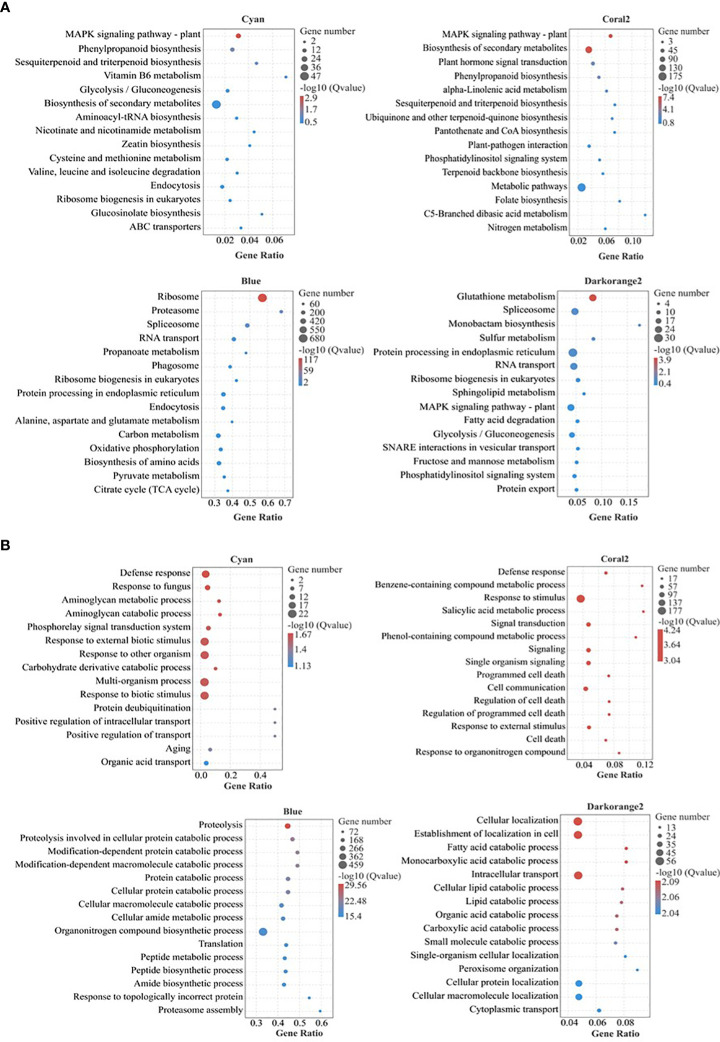
Bubble diagram of KEGG and GO pathway enrichment of selected key modules. **(A)** KEGG pathway enrichment; **(B)** GO pathway enrichment (Biological process); Gene Ratio, the ratio of the number of genes enriched to each GO term to the total number of genes analyzed by GO. Pathways meeting the condition of Q value ≤ 0.05 were defined as significantly enriched pathways.

KEGG Pathway enrichment analysis (**Figure 5A**; **Table S4**) showed that the highly enriched pathways in the Cyan module included the plant MAPK signaling pathway (ko04016), phenylpropane biosynthesis (ko00940), sesquiterpene and triterpene biosynthesis (ko00909), vitamin B6 metabolism (ko00750), glycolysis/gluconeogenesis (ko00010), biosynthesis of secondary metabolites (ko01110), cysteine and methionine metabolism (ko00270), etc. The highly enriched pathways in the Coral2 module included the plant MAPK signaling pathway (ko04016), biosynthesis of secondary metabolites (ko01110), plant hormone signal transduction (ko04075), phenylpropane biosynthesis (ko00940), alpha-linolenic acid metabolism (ko00592), sesquiterpene and triterpene biosynthesis (ko00909), ubiquinone and other terpenoid-quinone biosynthesis (ko00130), metabolic pathways (ko01100), and nitrogen metabolism (ko00910). The highly enriched pathways in the Blue module included ribosome (ko03010), proteasome (ko03050), spliceosome (ko03040), RNA transport (ko03013), propanoate metabolism (ko00640), protein processing in endoplasmic reticulum (ko04141), alanine, aspartate and glutamate metabolism (ko00250), carbon metabolism (ko01200), oxidative phosphorylation (ko00190), biosynthesis of amino acids (ko01230), pyruvate metabolism (ko00620), citrate cycle (TCA cycle) (ko00020), etc. The highly enriched pathways in the Darkorange2 module were glutathione metabolism (ko00480), sulfur metabolism (ko00920), spliceosome (ko 03040), protein processing in endoplasmic reticulum (ko04141), RNA transport (ko03013), ribosome biogenesis in eukaryotes (ko03008), plant MAPK signaling pathway (ko04016), fatty acid degradation (ko00071), glycolysis/gluconeogenesis (ko00010), etc.

GO enrichment analysis (**Figure 5B**; **Table S5**) (only biological processes were analysed here) showed that the highly enriched biological processes in the Cyan module included defence response (GO:0006952), aminoglycan catabolic process (GO:0006026), aminoglycan metabolic process (GO:0006022), phosphorelay signal transduction system (GO:0000160), response to external biological stimulus (GO:0043207), response to other organism (GO:0051707), carbohydrate derivative catabolic process (GO:1901136), response to biotic stimulus (GO:0009607), positive regulation of intracellular transport (GO:0032388), positive regulation of transport (GO:0051050), etc. The biological processes highly enriched in the Coral2 module included defense response (GO:0006952), response to stimulus (GO:0050896), salicylic acid metabolic process (GO:0009696), benzene-containing compound metabolic process (GO:0042537), signal transduction (GO:0007165), phenol-containing compound metabolic process (GO:0018958), response to external stimulus (GO:0009605), hormone-mediated signaling pathway (GO:0009755), cell response to endogenous stimuli (GO:0071495), salicylic acid mediated signaling pathway (GO:0009863), response to salicylic acid (GO:0009751), etc. The biological processes highly enriched in the Blue module included proteolysis (GO:0006508), protein catabolic process (GO:0030163), cellular macromolecule catabolic process (GO:0044265), organonitrogen compound metabolic process (GO:1901564), macromolecule catabolic process (GO:0009057), protein localization (GO:0008104), etc. The biological processes highly enriched in the Darkorange2 module included cellular localization (GO:0051641), intracellular transport (GO:0046907), establishment of cellular localization (GO:0051649), fatty acid catabolic process (GO:0009062), cellular lipid catabolic process (GO:0044242), organic acid catabolic process (GO:0016054), carboxylic acid catabolic process (GO:0046395), peroxisome organization (GO:0007031), and protein targeting to peroxisome (GO:0006625).

### Identification of potassium-regulated hub genes of cotton root salt tolerance

**Figure 6:**
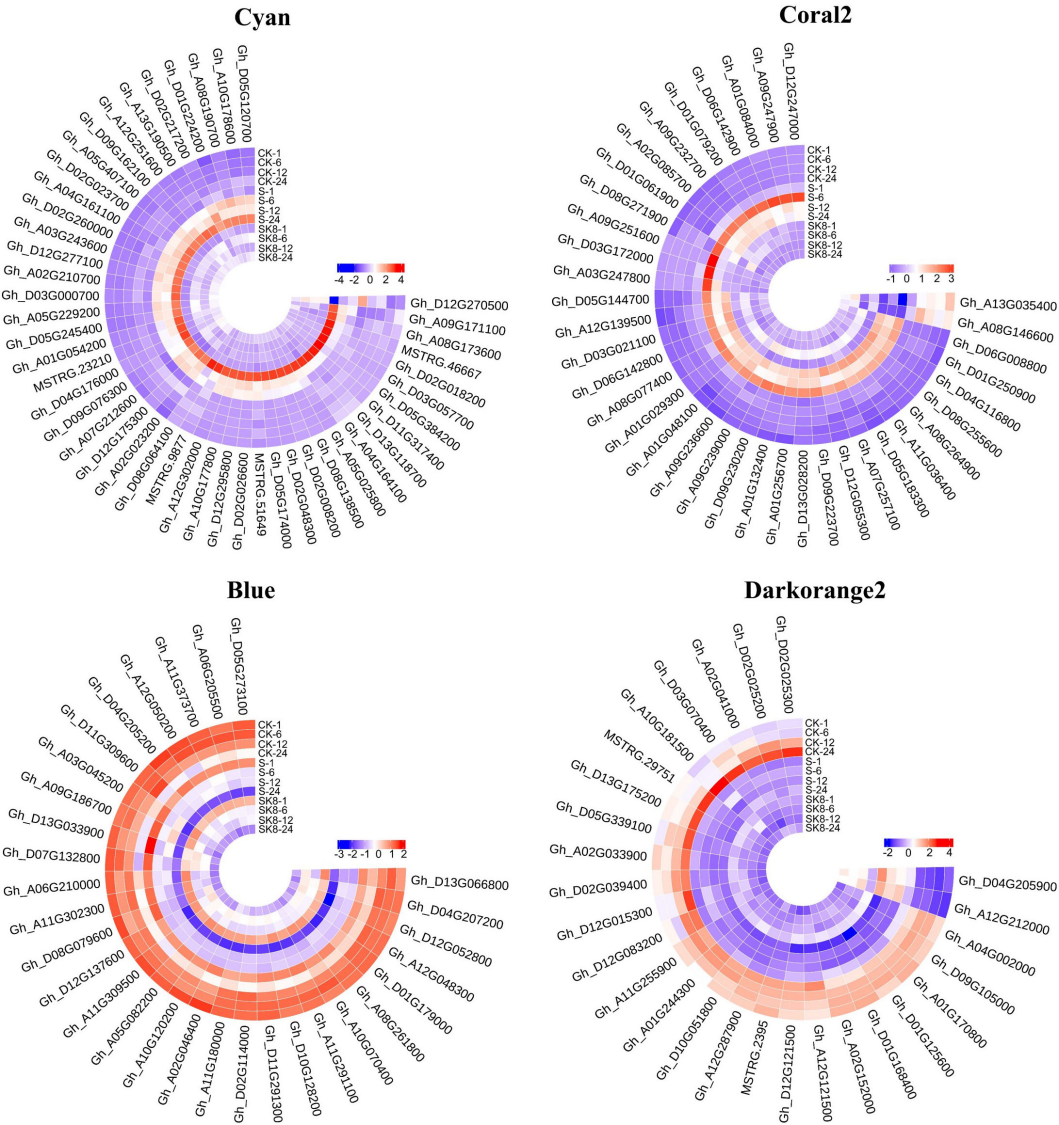
Heatmap of hub genes and their associated gene expression patterns in key modules. The color in the heatmap indicates the gene expression. Red represents high expression, and blue represents low expression.

In general, the expression patterns and functional enrichment of genes in each key module were significantly different after gene classification using WGCNA, which was in line with our expected goal. It is helpful for us to explore the key salt tolerance genes among a large number of differential genes and to analyse the regulatory mechanism of potassium on salt adaptation of cotton roots. After screening KEGG pathways of each module with Qvalue < 0.05 as the threshold, this study identified the plant MAPK signaling pathway and phenylpropanoid biosynthesis in Cyan module, the plant MAPK signaling pathway in Coral2 module, glutathione metabolism pathway in Darkorange2 module and TCA cycle in Blue module as the key pathways. The genes enriched in the key pathways of each module were sorted according to the order of gene connectivity from high to low. The top 5 genes with gene connectivity were regarded as hub genes, as detailed in **Table 1**. **Figure 6** shows the expression patterns of the hub genes and associated candidate genes of each key module.

**Table 1:** Details of hub genes in key co-expression modules.

Module	Gene_ID	Description
Cyan	*Gh_D12G270500*	Abscisic acid receptor PYL
	*Gh_D04G176000*	Peroxidase POD
	*Gh_D12G175300*	Peroxidase POD
	*Gh_D09G162100*	Peroxidase POD
	*Gh_A09G171100*	Peroxidase POD
	*Gh_D02G217200*	Peroxidase POD
Coral2	*Gh_D01G079200*	Mitogen-activated protein kinase kinase kinase
	*Gh_A13G035400*	Serine/threonine-protein kinase SRK2A
	*Gh_A08G146600*	Abscisic acid receptor PYL
	*Gh_D08G271900*	Protein phosphatase 2C
	*Gh_A01G084000*	Mitogen-activated protein kinase kinase kinase
Blue	*Gh_A11G309500*	ATP-citrate synthase
	*Gh_A10G120200*	Succinate dehydrogenase
	*Gh_D11G309600*	ATP-citrate synthase
	*Gh_A02G046400*	Malate dehydrogenase
	*Gh_D13G066800*	Probable succinyl-CoA ligase
Darkorange2	*Gh_D02G039400*	Glutathione S-transferase GST
	*Gh_D05G339100*	Glutathione S-transferase GST
	*Gh_A01G244300*	Glutathione S-transferase GST
	*Gh_A02G033900*	Glutathione S-transferase GST
	*Gh_D13G175200*	Glutathione S-transferase GST

### Construction of a potassium-regulated salt tolerance network in cotton roots

**Figure 7:**
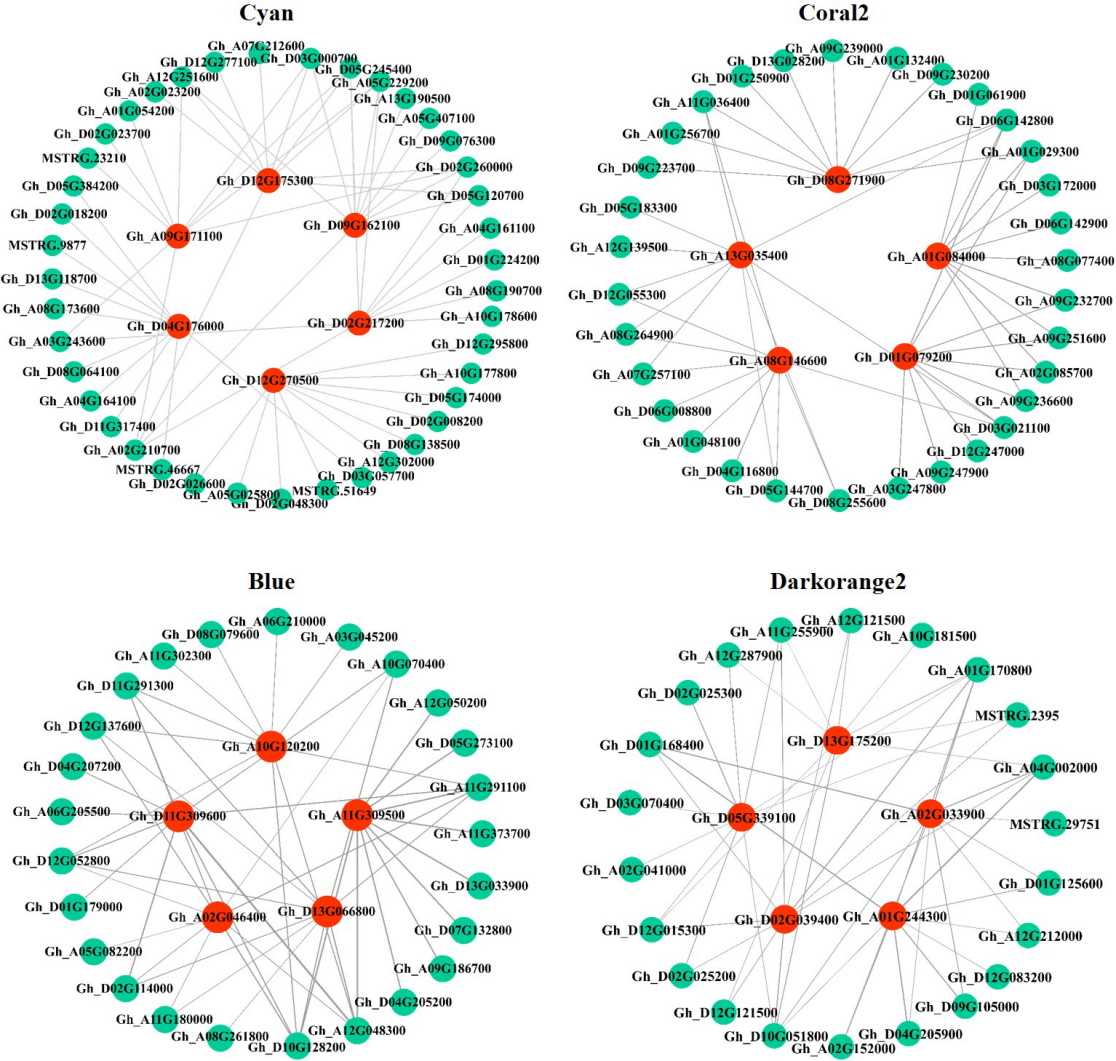
Regulatory network diagram of hub genes and associated genes in key modules. Red represents hub genes, blue−green represents the top 10 genes associated with hub genes.

In this study, the top 10 genes in the key modules connected to the hub genes were identified as associated genes (**Table S6**). Forty-one associated genes were screened by the Cyan module, including *Gh_A08G190700*, *Gh_D05G245400*, *Gh_A05G229200*, *Gh_D05G120700*, and *Gh_A07G212600*, which encode ERF transcription factors, and *Gh_A10G178600* and *MSTRG.9877*, which encode MYB transcription factors, as well as some genes encoding hormone signaling- and secondary metabolism-related proteins or enzymes. Thirty-two associated genes were screened by the Coral2 module, including *Gh_A07G257100* encoding the WRKY transcription factor and *Gh_A09G247900* encoding the BHLH transcription factor, as well as some genes encoding transporters or enzymes related to secondary metabolism, protein processing, and amino sugar and nucleotide sugar metabolism. Twenty-five associated genes were screened by the Blue module, including some genes encoding proteins or enzymes related to secondary metabolism and ribosomal transport. Twenty-two associated genes were screened by the Darkorange2 module, including some genes encoding proteins or enzymes related to glutathione metabolism, secondary metabolism, carbon metabolism, and amino acid biosynthesis. The weighted gene co-expression network was drawn using Cytoscape_3.3.0 (**Figure 7**). Nodes in the network represent genes, and edges represent regulatory relationships between genes. The more connected genes there are, the more important the gene is in the interaction network.

### Validation of the hub genes by RT-qPCR

**Figure 8:**
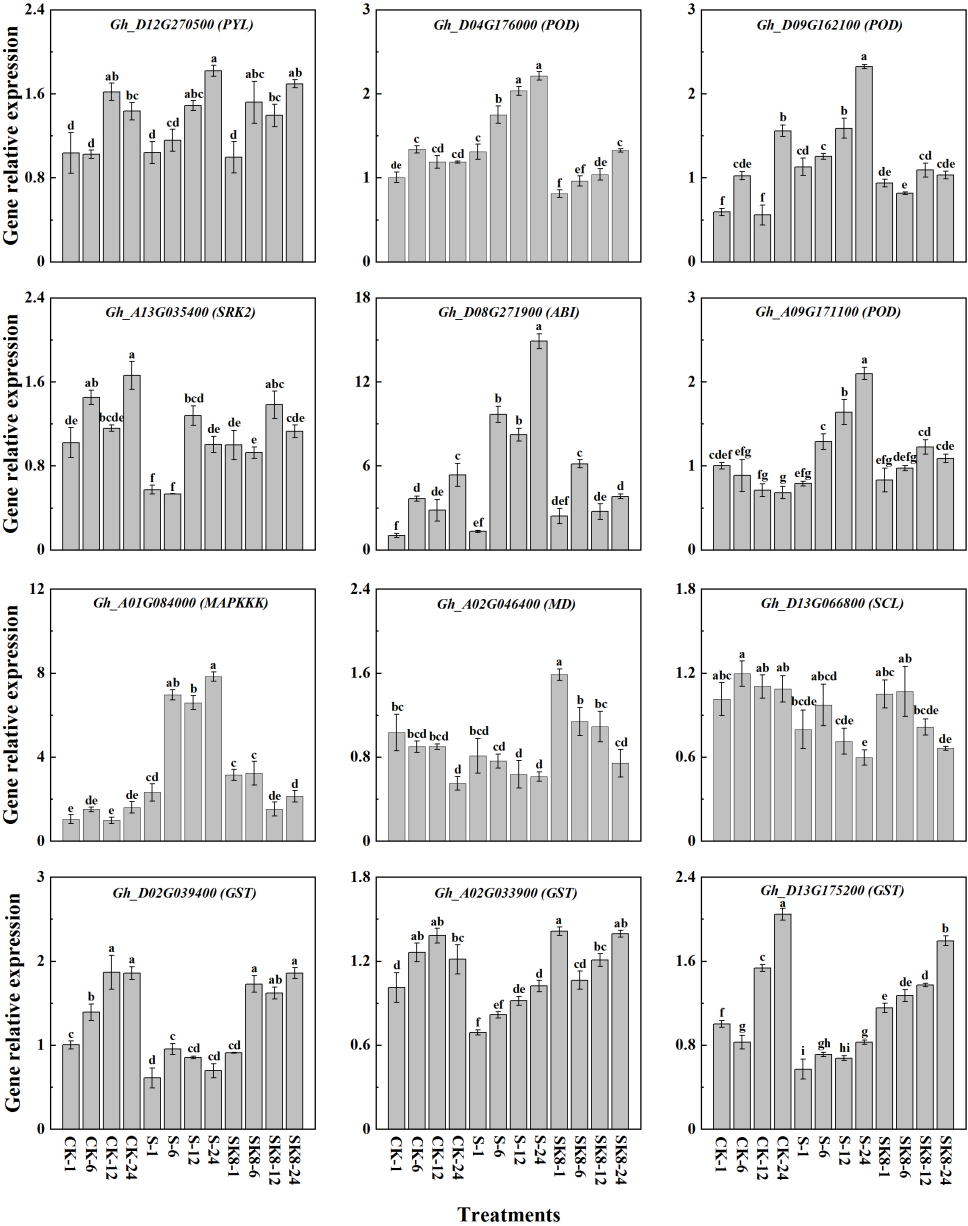
Validation of the hub genes by RT-qPCR. Different letters represent significant differences at the 0.05 level.

To verify the reproducibility and authenticity of the RNA-seq data, 12 hub genes with high expression in key modules were selected for qRT-PCR analysis (**Table S7**). As shown in **Figure 8**, qRT-PCR results of all 12 genes were consistent with the expression pattern of RNA-seq data. Genes significantly up-regulated in RNA-Seq data also exhibited an up-regulation in qPCR, and vice versa. These results also confirmed the reliability of the RNA-Seq data.

## Discussion

### WGCNA is a key strategy to reveal the mechanism by which potassium regulates salt adaptation in cotton

Cotton is one of the pioneer crops widely planted in saline-alkali areas, providing daily necessities such as natural fiber, plant protein and edible oil (Sharif et al., 2019). However, the expansion and long-term development of saline soil has directly threatened the productivity of crops, including cotton. Increasing the application of potassium fertilizer has been proven to be an important strategy to enhance the resistance of crops to stress (Ju et al., 2021), but the regulatory mechanism by which potassium fertilizer improves the adaptation of cotton roots to salt stress has not been fully explained. In recent years, the transcriptome has opened a new door for analysing and revealing the molecular mechanisms and biological processes of plant abiotic stress responses (Zhu et al., 2019). However, how to effectively mine and utilize the massive data obtained by RNA-Seq has always been a thorny problem for scientists. Differential expression analysis technology focusing on a single gene often misses a large amount of effective information. Therefore, WGCNA based on the overall gene expression patterns was proposed (Langfelder and Horvath, 2008). Currently, combinatorial analysis of RNA-Seq and WGCNA has emerged as a method to discover key genes and interactions that may be related to stress function (Leng et al., 2021; Ma et al., 2021). In this study, WGCNA was used to analyse the effective statistics and bioinformatics of an RNA-Seq data set, and the regulatory mechanism of potassium on the cotton root response to salt stress was analysed from the global gene expression pattern. By screening the transcriptome data of cotton roots treated with CK, S and SK8 for 1, 6, 12 and 24 h, 24395 high-quality genes were obtained and divided into 20 co-expression modules. Four key modules, Cyan, Coral2, Blue and Darkorange2, which are highly related to potassium regulation of cotton salt tolerance, were identified according to the expression pattern of module genes. Furthermore, KEGG Pathway enrichment analysis showed that the plant MAPK signaling pathway and phenylpropane biosynthesis pathway in the Cyan module, the plant MAPK signaling pathway in the Coral2 module, the glutathione metabolism pathway in Darkorange2 module and the tricarboxylic acid cycle (TCA cycle) pathway in the Blue module were the key pathways in the above modules. At the same time, 21 hub genes and 120 candidate genes highly associated with hub genes were selected from key modules. It is speculated that these genes may be the dominant factors regulating the adaptation of cotton to salt stress.

### Potassium enhances the salinity tolerance of cotton by regulating the MAPK signaling pathway

Plant salt tolerance is achieved through the combination of cellular, molecular and physiological metabolic reactions (Amirbakhtiar et al., 2021). Therefore, it is necessary to take measures to explore and improve the key components of salt tolerance regulation. Plants activate a complex set of signaling pathways in response to various abiotic threats. Among them, the MAPK signaling pathway is an important mechanism for plants to respond to external stimuli (Danquah et al., 2014; Yao et al., 2022. MAPKs are a group of intracellular signal transduction factors that transduces extracellular stimulation signals into plant cells through three-layers protein kinases, namely, MAPK kinase kinase (MAPKKKs), MAPK kinase (MAPKKs) and MAPKs (Coulthard et al., 2009; Gehart et al., 2010). Activation of MAPK elements and downstream genes may affect plant stress resistance (Wei et al., 2022). In the present study, the MAPK signaling pathway was significantly enriched in the Cyan and Coral2 modules, and hub genes related to abscisic acid receptor (PYL)-, protein phosphatase (PP2C)-, serine/threonine protein kinase (SnRK2)-, and protein kinase kinase kinase (MAPKKK17/18) were up-regulated by salt stress. In contrast, potassium treatment down-regulated the expression of these genes. There has been evidence that the combination ABA and PYL can alleviate the inhibition of SnRK2 by PP2C, activate SnRK2, induce plant stress response, and participate in the downstream MAPK signaling pathway (Liu et al., 2022). The expression of all MAPKs in chickpea was significantly increased under abiotic stress (Singh et al., 2017). Moreover, overexpression of *OsMAPK33* reduced the tolerance of rice to salt stress (Lee et al., 2011). These evidence indicated that MAPKs played a negative regulatory role in plant resistance to abiotic stress. Consistent with previous conclusions, this study found that salt stress activated the expression of key elements in MAPK signaling pathway, while potassium application reversed the activation effect by reducing the expression of MAPKs and maintaining the stability of MAPK signal transduction system, which may be one of the possible reasons why potassium alleviates salt damage in cotton.

### Potassium improved the salt tolerance of cotton by promoting the TCA cycle

Increasing the energy produced by respiration is one of the survival strategies for plants to adapt to various stress environments, such as glycolysis, amino acid metabolism, and the TCA cycle. (Kumari et al., 2015; Chen et al., 2019; Bandehagh and Taylor, 2020; Guo et al., 2022a). In plants, the TCA cycle is a pivotal link between energy metabolism with carbon and nitrogen metabolism (Nunes-Nesi et al., 2013), providing adenosine triphosphate (ATP) and reducing agents for ion efflux, osmotic agent synthesis and reactive oxygen species removal (Munns & Tester, 2008; Che-Othman et al., 2020). Citric acid, aconitic acid, α-ketobutyric acid, fumaric acid and malic acid are the main intermediates in the TCA cycle and important mediators of material metabolism and energy transformation (Li et al., 2022). Previous studies have shown that salinity inhibits the activities of photosynthetic carbon metabolism and the TCA cycle. Salt stress resulted in decreased concentrations of most intermediates associated with the TCA cycle in maize hybrids (Richter et al., 2015) and *Karelinia caspia* (Guo et al., 2022b). The researchers suggest that maintaining the TCA cycle helps plants survive stress conditions such as high temperature (Li et al., 2016), drought (Li et al., 2017b) and salt stress (Che-Othman et al., 2020). Salt-tolerant soybean varieties have higher amino acid accumulation rate and faster TCA cycle activity (Jin et al., 2021). Some scholars also pointed out that wild soybean (Li et al., 2017a) promoted its energy metabolism and intermediate metabolite levels under salt-alkali stress by increasing TCA cycle activity, thus enhancing salt tolerance. In the present research, the TCA cycle was enriched in the Blue module, where the hub genes encoding ATP-citrate synthase ACLY, succinate dehydrogenase SDH, malate dehydrogenase MDH and succinyl CoA ligase SCUD were significantly down-regulated by salt stress, but significantly up-regulated after potassium application. The above results indicated that salt stress negatively regulated the TCA cycle in cotton roots, while potassium application activated the activity of the TCA cycle, which provided more carbon source and energy for the root response to salt stress, accelerated the physiological and metabolic response, and further enhanced the adaptability of cotton to salt stress.

### Potassium enhanced the salt tolerance of cotton by activating glutathione metabolism

Many inducible genes have been studied in plants under abiotic stress, including transcription factors (Erpen et al., 2018), protein kinases (Ye et al., 2017) and cytoprotective enzymes (Hanaka et al., 2018). Glutathione S-transferases (GSTs) are a group of multifunctional protective cellular enzymes that reduce ROS production by catalyzing the binding reactions of glutathione to various cytotoxic substrates (Hao et al., 2021). The expression and activity of GST genes were positively correlated with plant stress resistance (Cao et al., 2022). Several previous studies have identified the important role of GSTs in plant stress resistance. For example, transgenic plants that overexpressing GST improved the drought tolerance of *Arabidopsis thaliana* by enhancing ROS scavenging ability (Wang et al., 2012; Xu et al., 2015). *CsGSTU8* plays a positive role in drought resistance of camellia (Zhang et al., 2021). Overexpression of GSTs in tobacco (Roxas et al., 2000) and rice (Takesawa et al., 2002) increased the resistance of transgenic plants to high temperature, low temperature and salt concentration. Kumar et al. (2013) reported that overexpression of *OsGSTL2* enhanced plant resistance to cold, osmotic stress and salinity stress. Our results showed that the glutathione metabolism pathway was significantly enriched in the Darkorange2 module. Five hub genes in this pathway all encoded glutathione S-transferase GST, and were down-regulated by salt stress but up-regulated by potassium application treatment. Enhanced stress capacity is accompanied by up-regulation of antioxidant genes and increased activity of antioxidant enzymes such as GSTs (Hayes et al., 2005). The results of this study indicated that salt stress negatively regulated glutathione metabolism of cotton roots, which may lead to the outbreak of ROS in roots, resulting in salt damage. Potassium application increased the expression of GST gene, accelerated the clearance of ROS, improved the salt tolerance of cotton, and reduced the occurrence of salt damage. This conclusion was consistent with previous reports in tobacco (Du et al., 2019) and soybean (Ji et al., 2010; Chan and Lam, 2014).

## Conclusion

**Figure 9:**
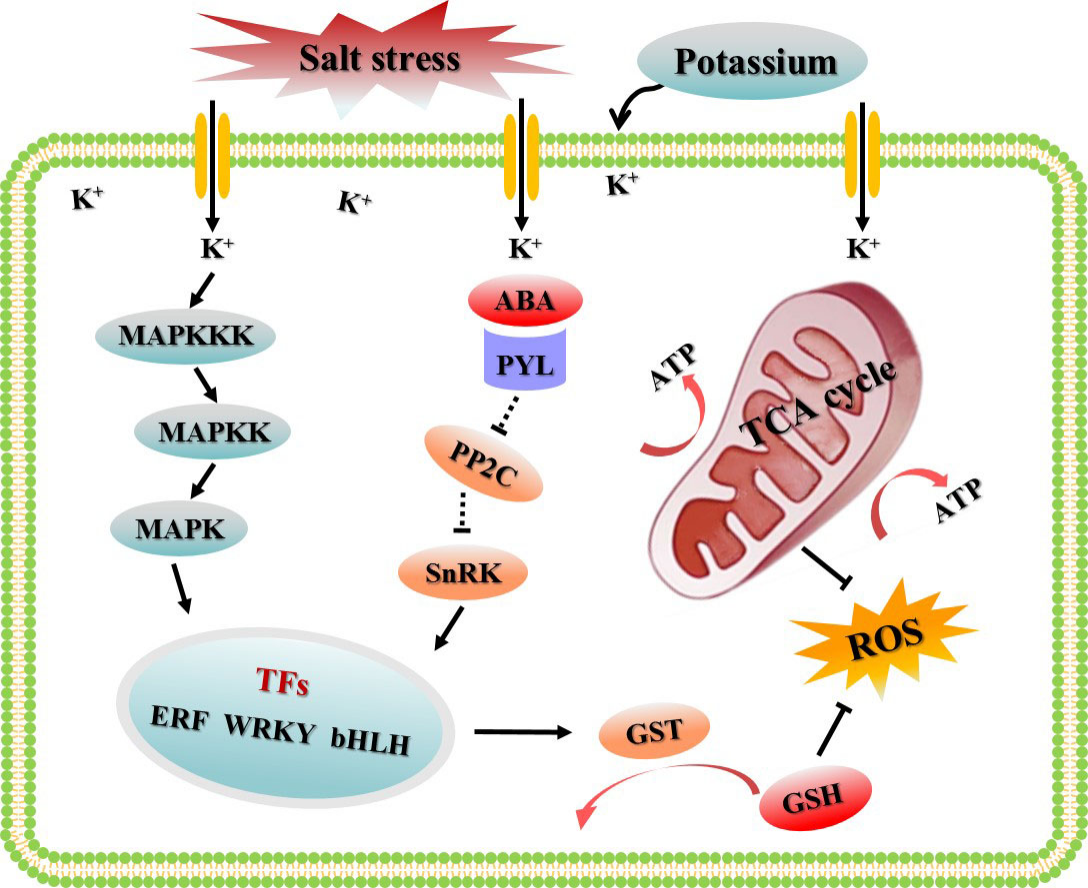
Schematic diagram of the regulatory mechanism by which potassium improves the salt adaptability of cotton roots based on WGCNA.

To sum up, in this study, the appropriate potassium application rate to alleviate salt damage of cotton was selected based on different K^+^/Na^+^ ratios, and a gene co-expression network based on WGCNA were constructed using the transcriptome data sets treated with CK, S, and SK8. The highlight of this study was to screen the key modules and hub genes of potassium to improve the salt adaptability of cotton roots based on the global gene expression. We proposed a hypothetical model to explain the regulatory mechanism of potassium on cotton root adaptation to salt stress (**Figure 9**). Network analysis showed that the MAPK cascade signaling pathway, TCA cycle and glutathione metabolism pathway were the key biological pathways for potassium to improve salt adaptability in cotton roots. In this study, 21 key genes and 120 key candidate genes were identified based on gene connectivity, which may be potential targets for potassium regulation of salt tolerance in cotton. Although the results of this study have some limitations, further experimental studies are needed to verify its function in regulating salt adaptation. However, these results provide reliable guidance for further research on the molecular mechanism and the digging of key genes of potassium promoting salinity adaptability in cotton and other plants.

## Data availability statement

The datasets presented in this study can be found in online repositories. The names of the repository/repositories and accession number(s) can be found below: Bio project accession number: PRJNA919499.

## Author contributions

FJ, ZZ, and BC were responsible for the experimental design. FJ, LS, and JP performed the experiments. FJ, HB, WZ and BC prepared the manuscript and coordinated its revision. CX and ZW and HY read and revised the manuscript. All authors contributed to the article and approved the submitted version.
